# Books Reviewed

**Published:** 1862-09

**Authors:** 


### BOOKS REVIEWED.

*A Practical Treatise on the Diseases of the Heart and Great Vessels, including the Principles of Physical Diagnosis, by* WALTER HAYLE WALSHE, M. D., *Fellow of the Royal College of Physicians; Professor of the Principles and Practice of Medicine and of Clinical Medicine in University College, London; Consulting Physician to the Hospital for Consumption. A new American, from the third revised and much enlarged London edition. Philadelphia:* BLANCHARD & LEA, 1862.

Our author gives first the Clinical Topography of the Heart and great Vessels in health, thus commencing at the foundation, the true starting point of correct observation. This is also embellished by a very fine illustrative cut showing the position and relations of the various organs under consideration, and adding very much to the correct understanding of the whole subject. Weight, size and measurement in health are also given as obtained by extensive and careful investigation.

Clinical Physical Examination of the Heart and Blood Vessels, constitute part first of the volume, including the fullest directions and explanations of the methods, viz: Inspection, application of the hand, palpation, mensuration, percussion and auscultation.

Part II is upon Diseases of the Heart and Great Vessels. The introductory remarks we copy for the consolation of physicians who have difficulty in some cases, in positively distinguishing organic from functional disease of that organ.

"Diseases of the heart, as of all other organs in the body; are divisible into two classes; in the one, as far as can be discovered, the dynamics of the organ alone are at fault; in the other, structural change is more or less apparent. Cardiac affections are hence dynamic and organic.

SECTION I.—DYNAMIC DISEASES OF THE HEART.—The different perversions of the dynamics of the heart which are known clinically, may exist in association with structural disease, as well as independently of this The distinction of the two conditions—the associated and the unassociated—sometimes simple enough, often proves in actual practice far from easy.

A review of some of the more important of the alleged rules for distinguishing the two classes will at once show their clinical insignificance. The inconstancy of the symptoms of dynamic, and the constancy of those of organic ailment, are strongly dwelt on, for example; but all the subjective, and many of the objective, symptoms may disappear temporarily in cases even of extensive organic disease. Such disappearance with ensuing recurrence may even take place several times. The existence of secondary changes, such as subcutaneous œdema, and congestion of the lung, commonly proves the cardiac affection to be organic; but not always, for spanæmia, added to nervous palpitation, may induce œdema. If exercise relieve a disturbed heart, its affection is pronounced to be dynamic only; if movement increase the suffering, organic. This proposition might lead to an incorrect impression; for, it is certain, if spanæmia co-exist with perverted action, exercise may be unbearable. If, in the intervals of attacks of disturbed action, the force and rhythm of the pulse and heart are natural, those attacks are said of necessity to be functional—an error; for the most perfect tranquility of the organ *may* exist, from time to time, though its texture is seriously unsound. It has been taught that inability to bear particular postures, more particularly that of decumbency on the left side, in cases of disordered action of the heart, shows that organic mischief exists. Often true, this proposition occasionally proves false; women with spanæmia, palpitating, though perfectly sound, heart, and left intercostal neuralgia, can rarely endure the posture in question.

Singularly enough, the amount of local suffering entailed by disturbance wholly, or in the main, dynamic is often greater—is, in the mass of cases, greater—than that produced by actual organic disease. The patient with grave organic mischief, that may kill him at any moment, say, marked aortic regurgitation, may be so free from cardiac suffering, as to express irritation that his heart should be made the subject of examination; another individual oppressed with disturbed rhythm and innervation of his structurally healthy heart, dependant on flatulent distension of the stomach, refuses to be persuaded of his freedom from mortal cardiac mischief. As a rule, apprehension is not often excited in the subject of structural disease of the heart, until the secondary and distant evils of the local morbid changes, whatever they be, begin to make themselves felt.

The truth is, careful and repeated physical examination alone can justify a positive opinion on this question; and even this aid will occasionally fail. For, in the first place, the mere existence of certain abnormal physical signs in a palpitating heart does not warrant the assumption that textural change exists. Thus a basic systolic murmur may be simply anæmic—it may by possibility arise from mere perverted action of the sigmoid valves—or depend simply on the unnatural force with which the blood-current rushes against the orifices of the great vessels. Again, a systolic murmur at the left apex of the heart may be generated by irregular action of the papillary muscles. Further, extension of the normal area of cardiac dullness even considerably to the right may depend on merely temporary obstruction of the blood's movement through the heart, leading to distension of its right cavities, and be wholly independent of any real dilatation or hypertrophy. Conversely, in the second place, the total absence of physical signs does not prove the heart to be in a perfect state of organic soundness; there are slight amounts of change in the heart's substance, of which the perverted signs (for, doubtless, such really exist) are beyond the penetration of the present day. Nor must the student forget that numerous conditions of adjacent structures may throw cardiac physical signs into the shade, and prevent the detection, unless extreme care be exercised, of any positive disease. Thus emphysematous or even bronchitic distension of the lung, causing this organ to overlap the heart to an abnormal extent, will prevent increased area of percussion-dulness by an enlarged heart; and horizontal conduction of amphoric note from the stomach or colon, may gravely deceive as to the actual outline of the heart. And such instances might readily be multiplied.

Assistance in cases of doubt may be obtained from diathetic peculiarities and co-existent diseased states. Functional disturbance of the heart is connected more or less constantly and markedly with the following conditions: *Perverted innervation*, as in cases of hysteria, spinal irritation, uterine and ovarian excitement, and various neuralgiæ, intercostal, dental, &c., *altered condition of the blood*, as in hemorrhage, anæmia, gout, chronic rheumatism, and chronic disease of the liver; *nervous exhaustion*, as from sexual excess, masturbation, or spermatorrhœa; *mechanical interference with the heart*, as when the stomach or intestines are disturbed with flatus; *certain poisonous influences*, as those of tobacco, green tea, and various diffusible stimulants. But none of these conditions are inconsistent with the presence of actual organic mischief.

Nevertheless, although cases occasionally occur in which it proves impossible to affirm or deny the presence of organic change (more especially, perhaps, of fatty atrophy,) the instances in which a painstaking observer is wholly baffled are really rare.

Now functional disorders of the heart, clinically recognized, being composed of various disturbances of the elementary dynamic properties of the heart, innervation and contractility, it seems likely to tend to a better understanding of those functional disorders, if we commence by an analysis of the demonstrable or possible perversions of the elementary properties. Clinically, however, this study is in its infancy; physiologically, even, our information is unsettled and incomplete. Hence, to a considerable extent, the statements I am about to lay before the student are to be accepted rather in the light of suggestion than of dogmatic assertion."

After a careful perusal of this book we feel more than ever our inability to make satisfactory review. It is a book, and subject for study, and no fair appreciation can be obtained without it. It would be a delight, if opportunity presented to examine the book and heart disease together, and we have often felt in reading it, the strongest desire to compare, see, hear, and feel what is so happily described. We shall not attempt to speak comparatively of this work on the Diseases of the Heart and Great Vessels; and as to pointing out its defects, we may as well frankly confess our inability to discover any, when we shall be left only to admire the careful observation which has been instituted and the value and distinctness of the conclusions which have been drawn. Positions are well taken and fully maintained, or candidly stated to be unsettled and unknown. We know of no author who has treated disease of the Heart more thoroughly or scientifically, or told more plainly and attractively all that is yet known upon this subject.

---

*Caries of Elbow Joint, Operation of Excision with recovery of useful arm; presented to Medical Society of State of New York, at its annual meeting,* 1862. *By* N. C. HUSTED, M. D., *New York City. Albany:* C. VAN BENTHUYSEN, 1862.

The patient was a young Miss, aged 14 years; of leuco-phlegmatic temperament, and scrofulous diathesis. She was operated upon 28th day of September, 1861. When the wound had entirely healed, passive motion of the elbow was practiced regularly. The patient was instructed to move the fore-arm and use the hand. At the end of two months succeeding the operation, the patient was able to dress herself, play the piano, and carry any ordinary scuttle filled with coal.

"Four months after the operation her general health was good, weighing twenty pounds more than before; elbow showed no tumefaction, no sinus, had in fact a healthy aspect; flexion and extension quite perfect; pronation and supination nearly so; shortening two inches."

This pamphlet contains also an interesting history of the operation of excision of the elbow joint. The case reported is an exceedingly interesting one, and a full description of the operation, after dressings and treatment, is given in detail, adding to the interest of the report. It seems to us that the results of the operation, are uncommonly satisfactory.

